# Consumer Stockpiling Across Cultures During the COVID-19 Pandemic

**DOI:** 10.1177/1069031X211037590

**Published:** 2022-06

**Authors:** Iman Ahmadi, Johannes Habel, Miaolei Jia, Nick Lee, Sarah Wei

**Keywords:** COVID-19, coronavirus, pandemic, stockpiling, culture, Hofstede

## Abstract

On March 11, 2020, the World Health Organization declared the COVID-19 (coronavirus) outbreak a pandemic. In the following days, media reports showed that consumers increasingly stockpiled groceries and household supplies. Interestingly, behavioral data show that this stockpiling exhibited considerable heterogeneity across countries. Building on cultural dimension theory, the authors theorize that this heterogeneity can be explained by countries’ cultural values: consumer stockpiling after the World Health Organization's announcement was more pronounced in countries whose residents show high uncertainty avoidance, low long-term orientation, low indulgence, and high individualism. The authors confirm these propositions using global mobility data from Google matched with country-level data on cultural values, pandemic reaction policies, and other key variables. This research note thereby integrates the previously disconnected literature on cultural dimension theory and consumer stockpiling in general, as well as provides new and significant knowledge about cross-cultural consumer behavior in crises. Furthermore, the authors provide actionable insights for international policy makers and business managers who aim to predict or control consumer stockpiling in future global crises to enhance consumer well-being.

In the early months of 2020, the evolving spread of COVID-19 kept the world in suspense. Having first been reported in late December 2019, the virus quickly spread around the globe (Kantis, Kiernan, and Bardi 2020). By early March, more than 118,000 people worldwide had been infected, and more than 4,000 had died. As a result, on March 11, 2020, the World Health Organization (WHO) declared COVID-19 a pandemic, calling on countries to “prepare and be ready” (Ghebreyesus 2020).

As concerns about the pandemic's possible impact began to grow worldwide, it became increasingly evident that retailers were struggling to cope with consumers’ stockpiling of basic groceries and household supplies (Charm 2020). As the *New York Times* pointed out, “If there's one image that captures the panic sweeping through the United States this week, it might be the empty store shelves where toilet paper usually sits” (Corkery and Maheshwari 2020). Similar reports appeared in media across the world (BBC News 2020; *The Guardian* 2020; Thurau 2020). Such stockpiling negatively impacts consumer well-being: consumers suffer from the unusually high cost and low availability of everyday essential commodities, leading to increased anxiety and reduced life satisfaction (Lufkin 2020).

Consumer stockpiling is also reflected in Google's COVID-19 Community Mobility Report (Google 2020): immediately after the WHO's announcement, consumers’ visits to grocery shopping destinations received a worldwide boost (see Panel A of Figure 1). Interestingly, this immediate increase in visits exhibited considerable heterogeneity across countries and was much more prominent in some countries (e.g., Luxemburg, Bulgaria) than others (e.g., Japan, Indonesia; see Panel B of Figure 1).

**Figure 1.**
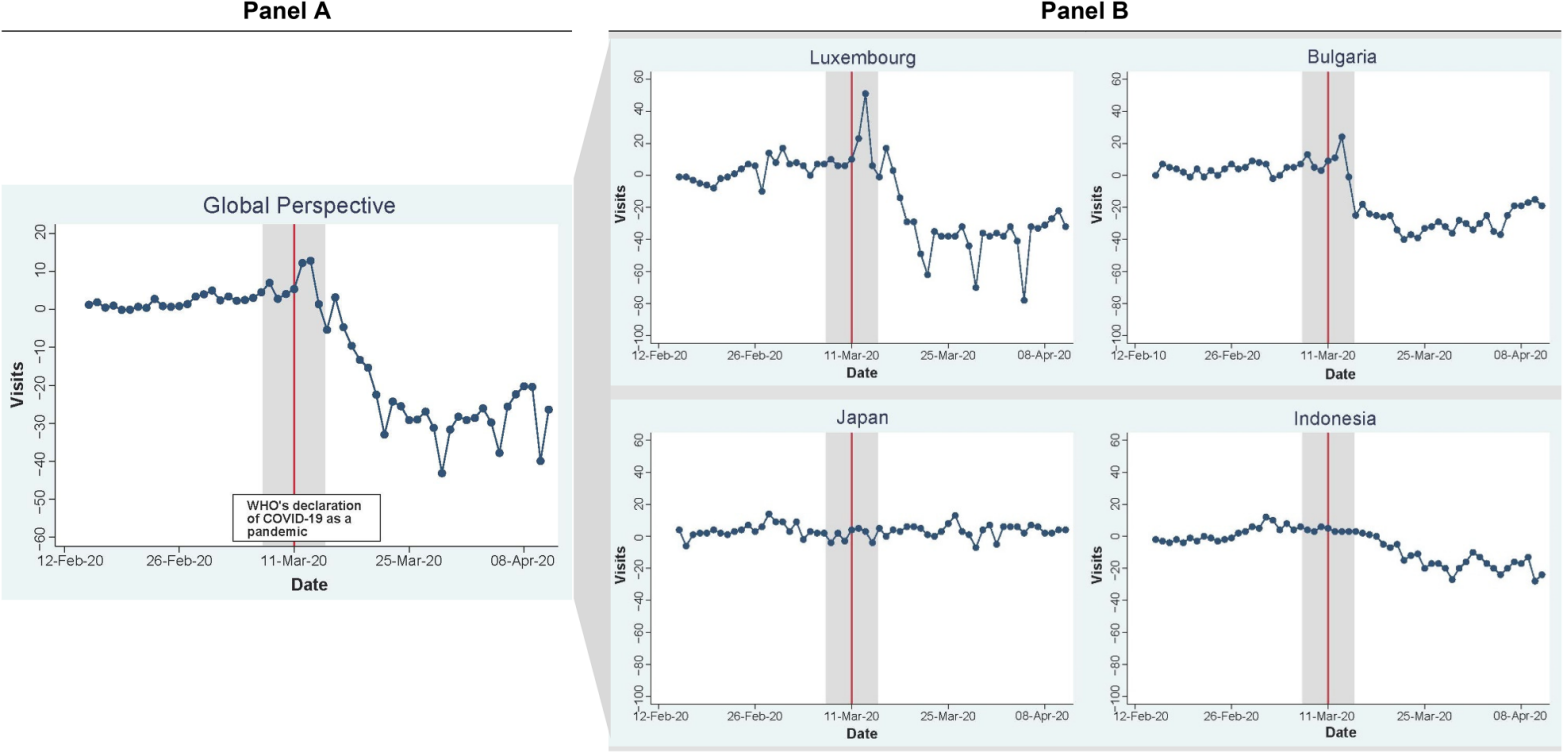
Global visits to grocery and pharmacy shopping destinations (and comparison with example countries) before and after the WHO's announcement of the COVID-19 outbreak.

*Notes*: The Global Perspective figure illustrates the average observed visits to shopping destinations such as grocery markets and food warehouses across 54 countries in our data set.

Notably, prior literature does not provide any explanation of why these intercountry differences should be observed. Research has explored how consumers react in response to natural disasters such as tornadoes. However, these studies focus on consumers within one culture (Baker and Hill 2013; Baker, Hunt, and Rittenburg 2007; Iacobucci 2019) and do not offer insights into how responses differ across cultures. In addition, research on consumer stockpiling has examined stockpiling as an outcome of price promotions (e.g., Bell, Chiang, and Padmanabhan 1999; Gupta 1988; Mela, Jedidi, and Bowman 1998). However, no prior work has examined stockpiling behavior across different nations, let alone as a reaction to a global crisis such as a pandemic.

In summary, little work appears to examine actual stockpiling behavior across nations in response to crises of any type, probably because of the historical challenges of accessing such data. However, recent advances in publicly accessible behavior data open new avenues for examining consumer behavior in many countries. We take advantage of such data to provide important new insights into cross-national consumer behavior—specifically, consumer stockpiling—in crises.

Our work therefore makes a significant contribution to international marketing, disaster, and stockpiling literature by developing a theory on cross-national differences in stockpiling in response to disasters. Specifically, by analyzing a global panel data set of consumer movement trends, we find strong empirical evidence to support that the heterogeneity in stockpiling following the WHO's announcement largely depends on cultural values across countries. Building on Hofstede's (2011) cultural dimension theory, we find that stockpiling is more pronounced in countries that exhibit high uncertainty avoidance, low long-term orientation, low indulgence, and high individualism.

With this research note, we hope to stimulate future research on cultural differences in consumer reactions to a global crisis. Further, our findings provide immediate and actionable policy and managerial implications for the effective management of stockpiling in response to emergency announcements, which can be crucially relevant to consumer well-being, particularly for the most vulnerable in our societies.

## Theoretical Background

We adopt Hofstede's (2011) cultural dimension theory to investigate the effect of national culture on stockpiling after the WHO's announcement. Hofstede's cultural dimension theory has been widely employed in international marketing research to explain differences in marketing communications and consumer behavior (e.g., Bahadir and Bahadir 2020; Dwyer, Mesak, and Hsu 2005; Eisingerich and Rubera 2010; Kim 2020; Pick and Eisend 2016). Further, recent studies in other fields have found that Hofstede's cultural values can explain differences in various national responses to the COVID-19 pandemic, such as collectivism increasing adherence to social distancing (e.g., Ashraf 2020; Im and Chen 2020; Kapitány-Fövény and Sulyok 2020; Yeung et al. 2021). We expect that Hofstede's value-based cultural dimensions (e.g., uncertainty avoidance) might also explain behavioral factors influencing stockpiling (Habel et al. 2020).

Hofstede (1983) notes that researchers should specify and focus on the most theoretically relevant cultural dimensions rather than always including all cultural dimensions in theory development, and this recommendation is commonly followed in existing international marketing research (Engelen and Brettel 2011; Griffith and Rubera 2014). As such, we focus on four cultural dimensions that exhibit a high theoretical fit with stockpiling after the WHO's announcement: uncertainty avoidance, long-term orientation, indulgence, and individualism.

### Uncertainty Avoidance

According to Hofstede (2011, p. 10), uncertainty avoidance “deals with a society's tolerance for ambiguity.” People in cultures characterized as high in uncertainty avoidance feel uncomfortable in unstructured situations that are “novel, unknown, surprising, and different from usual” (Hofstede 2011, p. 10). Consumers in these cultures are motivated to reduce ambiguity and rebuild structure, and thus, they prefer stability in their consumption (Erdem, Swait, and Valenzuela 2006). The WHO's announcement of COVID-19 as a pandemic highlighted the uncertainty that consumers faced, including the severity of COVID-19, possible disruptions of food production and supply (Fiondella 2020), and potential lockdowns that would limit consumers’ access to supplies (Secon, Frias, and McFall-Johnsen 2020). Consumers who live in countries typified by high uncertainty avoidance should thus have been particularly motivated to stockpile groceries to reduce the uncertainty in consumption caused by the COVID-19 pandemic.

Proposition 1: The increase in stockpiling following the WHO's announcement is more (less) pronounced for countries characterized as having high (low) uncertainty avoidance.

### Long-Term Orientation

Long-term orientation refers to the extent to which people emphasize future-oriented values that stabilize the structure of a society (De Mooij and Hofstede 2010; Hofstede and Bond 1988). People who are socialized in long-term-oriented cultures believe that important life events are likely to happen in the future and deemphasize immediate reaction (Hofstede 2011). They also put less weight on actions that reinstate their personal stability (De Mooij and Hofstede 2010). In line with this argument, prior research on intertemporal choice (Loewenstein and Thaler 1989) and self-control (Muraven and Baumeister 2000) has suggested that orientation toward the future prompts consumers to resist the temptation of spending for the present. The WHO's announcement is likely to be considered a short-term shock. Consumers in long-term-oriented countries should thus be less likely to strive to reinstate their personal stability through stockpiling of groceries and household supplies.

Proposition 2: The increase in stockpiling following the WHO's announcement is more (less) pronounced for countries characterized as having low (high) long-term orientation.

### Indulgence

Indulgence (vs. restraint) characterizes societies that value the freedom to gratify human desires and to enjoy life, which prompts consumers to face the world with an optimistic perspective (Hofstede 2011). These features seem likely to counteract the impact of crises such as the COVID-19 pandemic. By contrast, consumers who live in low-indulgence (or high-restraint) cultures hold a more pessimistic view and should thus be more likely to worry about potential stockouts of essentials. Moreover, consumers in low-indulgence cultures are more focused on practicalities (Hofstede 2011), which should lead to a stronger desire to stock up on essentials in the face of the pandemic.

Proposition 3: The increase in stockpiling following the WHO's announcement is more (less) pronounced for countries characterized as being low (high) in indulgence.

### Individualism

Hofstede (2011) defines individualism (vs. collectivism) as the degree to which people in a society are integrated into groups. A person in a culture characterized as high in individualism is “expected to look after him/herself and his/her immediate family” (Hofstede 2011, p. 10). We expect that high (vs. low) individualism renders people less likely to consider the potential negative effects of stockpiling on other community members. Moreover, people in high- (vs. low-) individualism societies care more about individual freedoms (Krause 2015) and would thus be more likely to ignore the government’s restriction measures (e.g., staying at home; Im and Chen 2020) and go out to buy supplies.

Proposition 4: The increase in stockpiling following the WHO's announcement is more (less) pronounced for countries characterized as being high (low) in individualism.

The four aforementioned dimensions are most likely to explain why consumers stockpile in response to a crisis. However, according to Hofstede (2011), cultures can be characterized along two additional dimensions (i.e., power distance^1^ and masculinity^2^), the influence of which on consumer stockpiling remains unclear. We do not form any propositions for these two dimensions. To account for the potential effects of these two dimensions, we controlled for both factors in our empirical model.

## Data Description

We used consumers’ country–day movement trends to grocery and pharmacy destinations (e.g., grocery markets, food warehouses, farmers’ markets, specialty food shops, pharmacies; hereinafter, “shopping destinations”) from February 15 to April 11, 2020, using the COVID-19 Community Mobility Report data set (Google 2020). Our analyses focused on the daily percentage change in visits^3^ to shopping destinations compared to the respective typical day of the week in early 2020 for 131 countries or regions. We matched this data set with data on countries’ cultural values, using Hofstede's (2015) six cultural value scores.

The decision to visit a shopping destination might be affected by the progression of the pandemic or lockdown policies in a country. We therefore controlled for two additional factors. First, we matched our country-level data set with the daily total number of identified cases and deaths due to COVID-19 (European Union Open Data Portal 2020). We operationalized the progression of the pandemic through the compound daily growth rate (CDGR) of COVID-19 cases and deaths. The CDGR of one week indicates the constant daily growth rate of today's number of new cases since the same day of the last week. The variable provides us with a comparable measure of the progression of the pandemic across countries.^4^ Second, we matched our country-level data set with the daily local government lockdown policies (OxCGRT 2021). We specified dummy variables indicating whether a country was under one of the following policies on each day: no lockdown policy, limited lockdown policy (i.e., recommended not leaving the house), moderate lockdown policy (i.e., allowed to leave the house only for essential trips, such as grocery shopping, exercise, etc.), and strict lockdown policy (i.e., not allowed to leave the house more than once a week).

In addition, to account for potentially intervening influences, we controlled for other variables that may impact our main results, including the gross domestic product (GDP) per capita as a proxy of a country's living standard (The World Bank 2019), the level of freedom available to journalists (as an indicator of communication of COVID-19-related news; Reporters Without Borders 2021), and the daily local government contact tracing policies (as a proxy for the number of COVID-19 tests; OxCGRT 2021). In particular, we included dummy variables indicating whether a country had no, limited (done for some COVID-19 cases), or a comprehensive (done for all COVID-19 cases) contact tracing program in place.

Overall, our data set covers 54 countries across the Americas, Asia, Europe, and Oceania over 57 days (i.e., N = 3,078 country/days = 54 × 57, where each observation represents a day in a country; for a summary of variables in our final data set, see Web Appendix Table W1; for a description and corresponding links of sources utilized to create our final data set, see Web Appendix Table W2).^5^ This substantial number of countries over time enables us to confidently trace differences in consumer visits to shopping destinations back to a country's cultural dimensions (Franke and Richey 2010).

## Methodology

To investigate how the WHO's announcement affects consumer visits to shopping destinations (hereinafter, “visits”) across countries with different cultural values, we specified the following equation:

$$\begin{array}{rl} {\left(\mathrm{VISIT}\right)}_{\mathrm{it}}= & \;\beta_{0}+[\beta_{1}\times \mathrm{HUA}I_{i}+\beta_{2}\times \mathrm{HLT}O_{i}+\beta_{3}\times \mathrm{HID}L_{i}\\ & +\beta_{4}\;\times \;\mathrm{HID}V_{i}+\beta_{5}\times \mathrm{HPD}I_{i}+\beta_{6}\times \mathrm{HMA}S_{i}]\\ & +[\beta_{7}\times \mathrm{LIMIT}\_\mathrm{NATL}D_{\mathrm{it}}+\beta_{8}\times \mathrm{MOD}\_\mathrm{NATL}D_{\mathrm{it}}\\ & +\beta_{9}\times \mathrm{STRICT}\_\mathrm{NATL}D_{\mathrm{it}}]\;\\ & +[\beta_{10}\times \mathrm{LIMIT}\_\mathrm{CONTTR}C_{\mathrm{it}}\\ & +\beta_{11}\times \mathrm{COMP}\_\mathrm{CONTTR}C_{\mathrm{it}}]\\ & +[\beta_{12}\times \mathrm{CDGR}\_7\_\mathrm{CAS}E_{\mathrm{it}}+\beta_{13}\\ & \times \mathrm{CDGR}\_7\_\mathrm{DEAT}H_{\mathrm{it}}]\\ & +\beta_{14}\times \mathrm{GDPP}C_{i}+\beta_{15}\times \mathrm{PRESSFD}M_{i}\\ & +[\delta \times \mathrm{WHOIMMEDIAT}E_{t}+\gamma \times \mathrm{WHOEXTEN}D_{t}\\ & +\theta \times \mathrm{WHOTREN}D_{t}+\mu \times \mathrm{TIM}E_{t}]+\epsilon_{\mathrm{it}}, \end{array}$$

where the dependent variable, VISIT_i_, is the daily percentage change in visits in country i; variables HUAI_i_, HLTO_i_, HIDL_i_, HIDV_i_, HPDI_i_, and HMAS_i_ represent the Hofstede cultural value scores of country i; LIMIT_NATLD_it_, MOD_NATLD_it_, and STRICT_NATLD_it_ represent whether country i was under a limited, moderate, or strict national lockdown policy on day t; LIMIT_CONTTRC_it_ and COMP_CONTTRC_it_ represent whether or not country i had a limited or comprehensive, respectively, contact tracing program in place on day t; CDGR_7_CASE_it_ and CDGR_7_DEATH_it_ represent the CDGR of one week for the number of cases and deaths, respectively, in country i on day t ∈ {1, 2, …, 57}; GDPPC_i_ represents GDP per capita in country i; and PRESSFDM_i_ is an index representing freedom available to journalists in country i.

In our equation, WHOIMMEDIATE_t_ and WHOEXTEND_t_ are pulse dummy and step dummy variables, respectively (Deleersnyder et al. 2002). Following the global trend in visits (see Panel A of Figure 1), we controlled for a two-day shock after the WHO's announcement; therefore, variable WHOIMMEDIATE_t_ equals 1 when t = 27 or t = 28, and 0 otherwise, while WHOEXTEND_t_ takes on the value of 1 when t ≥ 27 (i.e., any day after the WHO's announcement on March 11, 2020), and 0 otherwise. Intuitively, the pulse dummy, WHOIMMEDIATE_t_, controls for the two-day shock in visits due to the WHO's announcement while the step dummy, WHOEXTEND_t_, captures the average change in visits in the weeks following the WHO's announcement.

To avoid unobservable time-varying effects, we controlled for a deterministic (daily) trend variable, TIME_t_, which takes on values from 1 to 57, from February 15, 2020, until April 11, 2020. Similarly, WHOTREND_t_ controls for the growth rate of the trend curve after the WHO's announcement, which takes on the value of t − 27 + 1 when t ≥ 27, and 0 otherwise. Finally, ɛ_it_ represents the error term.

## Empirical Results

Immediately after the WHO's declaration of COVID-19 as a pandemic, countries on average experienced an abrupt boost in visits (see Panel A of Figure 1). This observation is confirmed in Model 1 of Table 1, which includes our results of the regression. Model 1 shows that in the two days after the WHO declared COVID-19 to be a pandemic, visits experienced a sudden increase (see the coefficient of WHOIMMEDIATE). We argue that this spike in visits indicates stockpiling—that is, the shift in purchase times before the expected time of the next purchase, and/or buying large quantities that enable consumers to increase their purchase intervals (Blattberg and Neslin 1990).

**Table 1.** Results for the Country's Cultural Values on Consumer Visits.

Variable	Model 1	Model 2	Model 3	Model 4
**Time-Related Variables**				
WHOIMMEDIATE	18.23** (.000)	2.99 (.785)	—	8.26 (.452)
WHOEXTEND	−7.64** (.000)	−14.37** (.001)	—	−18.61**(.000)
WHOTREND	−.73** (.000)	−.68** (.000)	−.68** (.000)	−.52** (.000)
TIME	.27** (.000)	.23**(.000)	.21** (.000)	.19** (.000)
**Interactions for Hofstede Cultural Dimensions**				
Uncertainty avoidance × WHOIMMEDIATE	—	.27** (.000)	.28** (.000)	.25** (.000)
Long-term orientation × WHOIMMEDIATE	—	−.21** (.006)	−.20** (.002)	−.25** (.001)
Indulgence × WHOIMMEDIATE	—	−.17* (.030)	−.16** (.007)	−.18* (.024)
Individualism × WHOIMMEDIATE	—	.26** (.001)	.26** (.000)	.23** (.002)
Power distance × WHOIMMEDIATE	—	.12 (.182)	.13* (.039)	.14 (.121)
Masculinity × WHOIMMEDIATE	—	−.06 (.342)	−.06 (.318)	−.06 (.346)
Uncertainty avoidance × WHOEXTEND	—	−.14** (.000)	−.17** (.000)	−.12** (.000)
Long-term orientation × WHOEXTEND	—	.25** (.000)	.20** (.000)	.30** (.000)
Indulgence × WHOEXTEND	—	.21** (.000)	.14** (.000)	.22** (.000)
Individualism × WHOEXTEND	—	−.02 (.434)	−.07** (.004)	−.02 (.429)
Power distance × WHOEXTEND	—	−.12** (.000)	−.19** (.000)	−.15** (.000)
Masculinity × WHOEXTEND	—	.02 (.519)	.02 (.438)	.02 (.494)
**Government Closure Policies (Base: None)**				
Limited lockdown policy	−9.52** (.000)	−9.02** (.000)	−9.34** (.000)	−8.96** (.000)
Moderate lockdown policy	−18.79** (.000)	−17.58** (.000)	−17.82** (.000)	−18.23** (.000)
Strict lockdown policy	−38.82** (.000)	−35.27** (.000)	−35.45** (.000)	−35.71** (.000)
**Contact Tracing (Base: None)**				
Limited contact tracing	1.69* (.014)	1.79** (.008)	1.67 (.102)	1.18 (.078)
Comprehensive contact tracing	.32 (.641)	.49 (.465)	.20 (.844)	.88 (.189)
**Controls (Other)**				
CDGR of cases over one week	−7.07** (.000)	−2.88 (.115)	−2.75 (.125)	—
CDGR of deaths over one week	−11.88** (.000)	−15.58** (.000)	−14.60** (.000)	—
Number of daily cases per capita	—	—	—	−5,730.43** (.000)
Number of daily deaths per capita	—	—	—	23,673.83 (.057)
GDP per capita	−.00* (.038)	−.00* (.045)	—	.00 (.392)
Press freedom	−.01 (.802)	−.01 (.840)	—	.00 (.922)
Controls for (six) Hofstede cultural dimensions	Yes	Yes	—	Yes
Controls for country fixed effects	No	No	Yes	No
Constant	−7.32** (.004)	−3.45 (.296)	3.81* (.047)	−2.15 (.511)
N	3,078	3,078	3,078	3,078
R^2^	.62	.65	.68	.65
Adj. R^2^	.62	.64	.68	.64

*p < .05.

**p < .01.

*Notes*: *p*-values are in parentheses.

Moreover, Model 1 of Table 1 shows that after the initial spike in visits following the WHO's announcement, the frequency of visits on average reduced significantly (see coefficient of WHOEXTEND). This dip in visits further confirms the existence of stockpiling behavior: the reduced visits indicate that consumers initially accumulated stocks and subsequently lived off these stocks (Web Appendix Figure W1 displays the predicted values from Model 1).

Overall, the abrupt increase in visits immediately after the WHO's announcement varies significantly across countries (see Panel B of Figure 1). To test our propositions that the impact of the WHO's announcement on stockpiling depends on a country's cultural values, we use a modified version of Model 1. We extended Model 1 by adding interaction terms between the country-level Hofstede cultural value scores and (1) consumers’ *immediate* visits, in the two days after the WHO's announcement (i.e., interactions with WHOIMMEDIATE), and (2) consumers’ *extended* visits in the weeks following the WHO's announcement (i.e., interactions with WHOEXTEND; see Model 2 of Table 1).

As Model 2 of Table 1 shows, consistent with Propositions 1 and 4, we found that consumers in countries of high uncertainty avoidance and high individualism engaged more in stockpiling in the two days after the WHO's announcement. Furthermore, in line with Propositions 2 and 3, for countries with high long-term orientation and high indulgence, consumers engaged less in stockpiling in the two days after the WHO's announcement (for further insights on [immediate] stockpiling across the four aforementioned cultural values, see Web Appendix Figure W2).

Our propositions are further confirmed by data on visits in the weeks following the WHO's announcement. We found that in countries with high uncertainty avoidance, visits in the weeks after the WHO's announcement decreased on average (see the negative coefficient of Uncertainty avoidance × WHOEXTEND in Model 2 of Table 1). As explained previously, such a decrease in visits is indicative of earlier stockpiling, because reduced visits are likely compensated by earlier large-basket purchases during consumers’ visits in the two days after the WHO's announcement. Similarly, we found shopping destinations in countries of low long-term orientation and low indulgence received fewer visits in the weeks after the WHO's announcement, whereas those in countries with high individualism received no change in visits. In summary, these results confirm our Propositions 1–3. We did not find a significant decrease in visits in countries with high individualism in the weeks following the WHO's announcement. One possibility would be that people in high-individualism countries were less likely to follow governments’ restriction measures (Im and Chen 2020) and might still go out to shopping destinations in the weeks following the WHO's announcement.

Regarding the remaining two cultural values, we did not find strong evidence of moderating effects. Specifically, Model 2 of Table 1 shows that neither power distance nor masculinity affects visits to shopping destinations in the two days after the WHO's announcement.

Regarding the remaining control variables, we found that visits decreased in countries with stricter lockdown measures, which provides support for the desired outcome of such decisions by local governments. Furthermore, our analysis reveals that local government lockdown policies have a stronger impact on stockpiling than the growth of COVID-19 cases in a country (i.e., the CDGR of one week for the number of cases or deaths due to COVID-19; see the results based on normalized variables in Table W3 of the Web Appendix), highlighting the effectiveness of such policies in decreasing consumers visits to shopping destinations.

Finally, we checked for the validity and robustness of our results in Model 2 of Table 1 in two ways. First, to control for country-invariant effects in our analysis, we introduced country fixed effects and ran a similar regression to that of Model 2 of Table 1 (see Model 3 of Table 1). Second, in Model 4 of Table 1, we checked the robustness of our results by replacing CDGR calculations of one week with the total daily number of cases and deaths per capita. Results from Models 3 and 4 of Table 1 replicate our main results in Model 2, which provides support for our propositions.

## Discussion and Conclusion

This research note provides important initial evidence that the WHO's declaration of the COVID-19 outbreak as a pandemic had an unintended consequence of driving an increase in consumer stockpiling and that the extent of this effect greatly depended on the cultural values across countries. Specifically, we found that consumers engaged in (immediate) stockpiling more in countries commonly associated with cultural values that motivate individuals to reduce uncertainty, to engage in myopic thinking that is short-term oriented, to emphasize restraint, or to put more weight on personal needs rather than the needs of society as a whole.

The results of this research note shed new light on international consumer behavior, specifically stockpiling, in response to a crisis. Previous international marketing research has mainly (unsurprisingly, it must be said) focused on economic crises, rather than issues such as global disasters and pandemics. Further, most preexisting work in this field examines consumer opinions, attitudes, or emotions in response to crises rather than actual cross-national consumer behavior. As such, our work provides an important early step in developing new knowledge in these areas.

Moreover, this research note contributes substantive new knowledge to Hofstede's cultural dimension theory by examining the impacts of Hofstede's cultural values on consumers’ reactions to a major crisis. Most prior research on cultural dimension theory focuses on how consumers construe value systems that guide everyday decisions (e.g., Hofstede 2011). Our findings suggest that cultural dimension theory also plays a pivotal role in influencing consumer stockpiling under the threat of a major crisis.

We also contribute to the stockpiling literature by showing that, in addition to being a consequence of price promotions (e.g., Gupta 1988), stockpiling can be triggered by the prominence of a crisis. Notably, the extent to which it is triggered is substantially determined by a country's uncertainty avoidance, long-term orientation, indulgence, and individualism. These findings provide cues for future research to further investigate how national cultures impact stockpiling, both in response to crises and other events.

In adding an international marketing dimension to the knowledge of stockpiling (and vice versa), our findings have important implications for nongovernmental organizations such as the WHO, policy makers, and managers regarding how to act to improve consumer well-being during a pandemic and even other global crises. First, our results help shed light on how public announcements (such as the WHO's declaration of COVID-19 as a pandemic) can act as an immediate trigger for stockpiling. Thus, policy makers and other relevant organizations (e.g., nongovernmental organizations) need to appreciate that their communication has direct implications for consumer purchase decisions, potentially even on a global scale. Importantly, these implications can include harmful unintended consequences on consumer well-being, with a particularly harmful effect on special consumer groups who have difficulties in visiting retail stores—as these stockouts appeared to fall most heavily on the vulnerable—as well as key workers such as nurses, who had less flexibility in timing their shopping trips (O’Reilly 2020).

**Figure 2.**
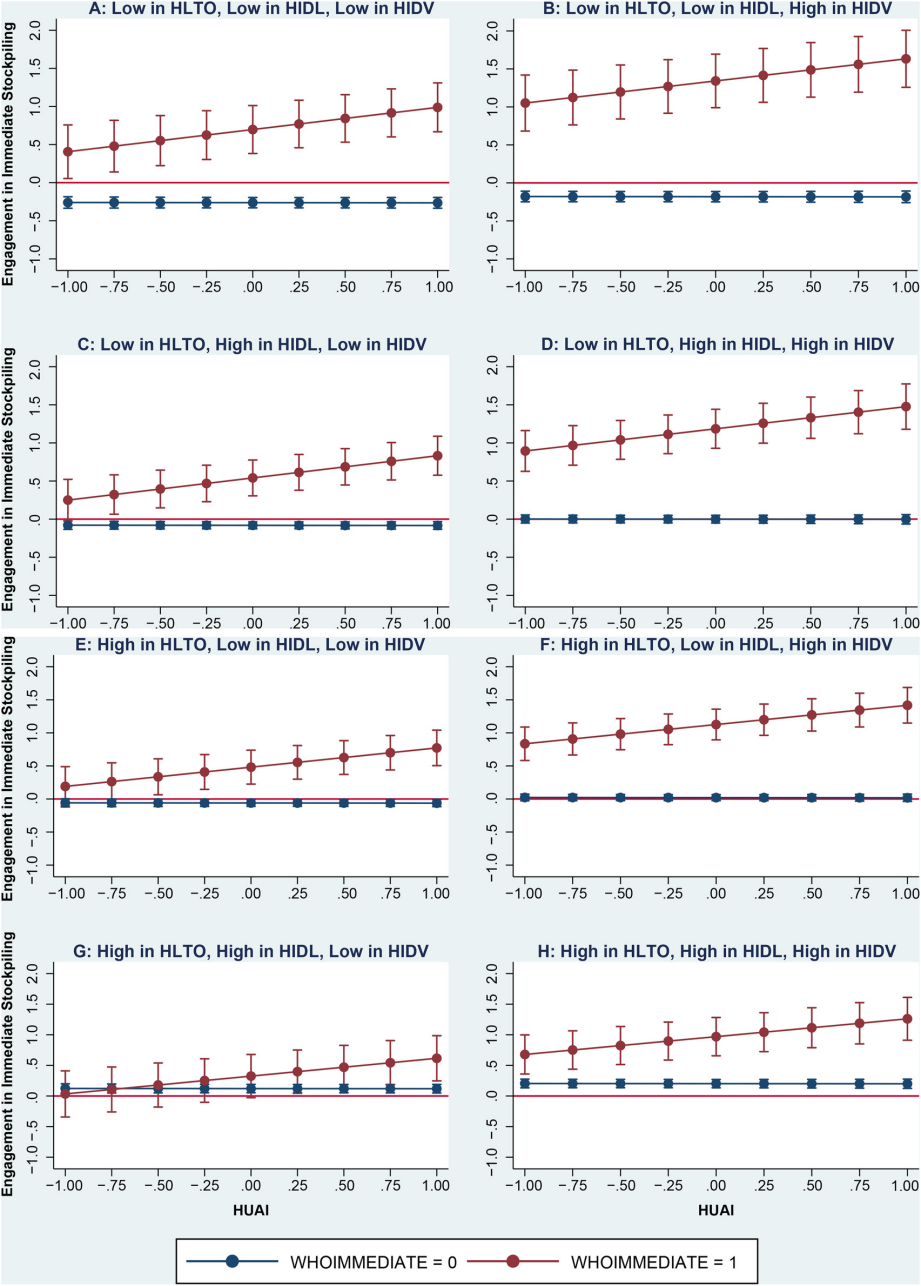
Comparison of immediate effect of different mixture of cultural values on consumers’ stockpiling due to the WHO's announcement of COVID-19 outbreak as a pandemic.

*Notes*: HUAI = uncertainty avoidance; HLTO = long-term orientation; HIDL = indulgence; HIDV = individualism. Vertical lines represent 95% confidence intervals; we used normalized variables (see Table W3 of Web Appendix); predicted values are estimated while setting (1) HLTO, HIDL, and HIDV scores to one standard deviation below and above their mean (for a country low and high in HLTO, HIDL, and HIDV, respectively) and (2) all other values at their means.

Second, policy makers and business managers from the whole supply channel (including manufacturers, distributors, and retailers) need to consider a country's culture when forming expectations of consumer purchase behavior in crises and taking preparatory actions to prevent stockouts. To provide policy makers and businesses with further insights, in Figure 2, Panels A–H, we plotted the degree of consumer stockpiling over different combinations of the four aforementioned cultural values (for the sake of comparability of results, we used normalized variables). As Figure 2 illustrates, and in line with our findings of Table 1, consumers in countries of relatively high uncertainty avoidance, low long-term orientation, low indulgence, and high individualism are most likely to react immediately and drastically to policy makers’ announcements (see Panel B of Figure 2). The opposite is true for consumers in countries of relatively low uncertainty avoidance, high long-term orientation, high indulgence, and low individualism (see Panel G of Figure 2). These findings can help policy makers and business managers make more informed decisions. For example, among countries with some cultural similarities (e.g., Bulgaria and Indonesia, which are long-term oriented, restrained, and collectivistic; see Panel E), consumers in countries with higher uncertainty avoidance (e.g., Bulgaria) tend to stockpile more than in those with lower uncertainty avoidance (e.g., Indonesia; in addition, compare Bulgaria and Indonesia’s figures in Panel B of Figure 1).

Third, our results guide policy makers on how to potentially limit stockpiling and thus improve societal well-being. For instance, consumers in cultures with high uncertainty avoidance might perceive stockpiling as a way to regain certainty and thus stockpile. In fact, across Hofstede's cultural dimensions, uncertainty avoidance has the highest immediate impact on stockpiling (see Web Appendix Table W3). Policy makers could use this insight to reassure and calm consumers through adequate communication.

Notwithstanding the contribution of this research note, we also recognize its limitations. Because we used the COVID-19 Community Mobility Report—which contains information on visits to grocery and pharmacy destinations using Google location history—a few shortcomings are worth noting. In particular, our data set (1) includes visits to pharmacies, in addition to groceries; (2) may be richer in more developed countries with higher availability of smart devices; and (3) may not fully capture consumer stockpiling via online ordering. It would be valuable for future research to use consumer shopping (online and offline) panel data from grocery retailers across the world to further examine our research question. Further research is also needed to examine the psychological process that drives consumers’ behavior in reactions to crises. Moreover, our research focuses on national cultures. Future research could investigate how economic and formal institutional factors (e.g., retail infrastructure and health care system) shape consumer behavior during a crisis.

## Supplemental Material

sj-pdf-1-jig-10.1177_1069031X211037590 - Supplemental material for Consumer Stockpiling Across Cultures During the COVID-19 Pandemic Click here for additional data file. Supplemental material, sj-pdf-1-jig-10.1177_1069031X211037590 for Consumer Stockpiling Across Cultures During the COVID-19 Pandemic by Iman Ahmadi, Johannes Habel, Miaolei Jia, Nick Lee and Sarah Wei in Journal of International Marketing

## Notes

1 According to Hofstede (2011), power distance refers to the extent to which the less powerful members accept and expect that power is distributed unequally. It is unclear why an acceptance or rejection of unequal power distributions would influence stockpiling after the WHO’s announcement. Therefore, we do not form a proposition on this cultural dimension.

2 According to Hofstede (2011), masculinity (vs. femininity) is related to the division of emotional roles between women and men. It is unclear why masculinity, or gender role difference in a society, would influence stockpiling after the WHO’s announcement. Therefore, we do not form a proposition on this cultural dimension.

3 The variable “visit” is a function of popular times, wait times, and visit duration, which captures the popularity of the shopping destinations (see https://support.google.com/business/answer/6263531).

4 A country’s CDGR over one week is calculated as (total number of cases on date D/total number of cases seven days earlier than date D)^(1/7)^ − 1.

5 The reason for a decrease from 131 to 54 countries is that some countries lack reliable information on cultural values, GDP, or the number of COVID-19 cases/deaths.
