# Supplementary material for: Consumer Stockpiling Across Cultures During the COVID-19 Pandemic
Source: J Int Mark. 2022 Jun;30(2):28–37. doi: 10.1177/1069031X211037590 (PMC9133904; doi:10.1177/1069031X211037590)
Supplement: sj-pdf-1-jig-10.1177_1069031X211037590 - Supplemental material for Consumer Stockpiling Across Cultures During the COVID-19 Pandemic [file sj-pdf-1-jig-10.1177_1069031X211037590.pdf]

Consumer Stockpiling Across Cultures During the COVID-19 Pandemic

Web Appendix

Iman Ahmadi
Assistant Professor of Marketing
Warwick Business School, University of Warwick,
Scarman Road, Coventry CV4 7AL, United Kingdom
Iman.Ahmadi@wbs.ac.uk

Johannes Habel
Associate Professor of Marketing
C.T. Bauer College of Business, University of Houston,
4750 Calhoun Road, Houston, TX 77204-6021, United States
jhabel@bauer.uh.edu

Miaolei Jia
Assistant Professor of Marketing
Warwick Business School, University of Warwick,
Scarman Road, Coventry CV4 7AL, United Kingdom
Miaolei.Jia@wbs.ac.uk

Nick Lee
Professor of Marketing
Warwick Business School, University of Warwick,
Scarman Road, Coventry CV4 7AL, United Kingdom
Nick.Lee@wbs.ac.uk

Sarah Wei
Assistant Professor of Marketing
Warwick Business School, University of Warwick,
Scarman Road, Coventry CV4 7AL, United Kingdom
Sarah.Wei@wbs.ac.uk

"These materials have been supplied by the authors to aid in the understanding of their paper.
The AMA is sharing these materials at the request of the authors."

Table W1 – Description and summary of variables

Variable	Description	N	Mean	SD	Min.	Max.
Movement trends						
VISIT	Percentage change in consumers’ visits to grocery and pharmacy destinations	3,078	−10.32	21.43	−95.00	59.00
Hofstede cultural dimensions						
HUAI	Uncertainty avoidance	54	67.28	23.10	8.00	112.00
HLTO	Long-term orientation	54	49.37	21.17	12.59	100.00
HIDL	Indulgence	54	48.94	21.48	.00	97.32
HIDV	Individualism	54	48.37	23.90	13.00	91.00
HPDI	Power distance	54	56.30	20.64	11.00	104.00
HMAS	Masculinity	54	48.69	21.23	5.00	110.00
National lockdown						
NATLD	The extent of the local government lockdown policy	3,078	.60	.92	.00	3.00
Contact tracing program						
CONTTRC	The extent of the local government contact tracing program	3,078	1.07	.81	.00	2.00
COVID-19						
CASEPC	Total no. of daily COVID-19 cases per capita	3,078	$1.67e^{-4}$	$4.69e^{-4}$	$.00e^{-4}$	$53.03e^{-4}$
DEATHPC	Total no. of daily COVID-19 deaths per capita	3,078	$.07e^{-4}$	$.29e^{-4}$	$.00e^{-4}$	$3.39e^{-4}$
CDGR_7_CASE	Compound daily growth rate of one-week COVID-19 cases	3,078	.13	.16	.00	1.41
CDGR_7_DEATH	Compound daily growth rate of one-week COVID-19 death	3,078	.07	.12	.00	.79
Economic and freedom of journalism situation						
GDPPC	GDP per capita (in $10,000)	54	3.03	2.54	.15	11.67
PRESSFDM	The level of freedom available to journalists	54	26.56	14.00	7.84	74.71

Table W2 – Description and corresponding links of datasets utilized for analysis in the main manuscript

Source	Description	Website
**A**	COVID-19 community mobility report dataset	https://www.google.com/covid19/mobility/
**B**	Daily number of COVID-19 cases and death	https://data.europa.eu/euodp/en/data/dataset/covid-19-coronavirus-data/resource/55e8f966-d5c8-438e-85bc-c7a5a26f4863
**C**	Hofstede cultural values	https://geerthofstede.com/research-and-vsm/dimension-data-matrix/
**D**	Information on countries GDP	https://data.worldbank.org/indicator/NY.GDP.MKTP.CD
**E**	Information on government lockdown policies and contact tracing program	https://www.bsg.ox.ac.uk/research/research-projects/coronavirus-government-response-tracker
**F**	World press freedom index	https://rsf.org/en

Note: If clicking on a link does not work, copy and paste the link directly into the browser.

Table W3 – Results for the country's cultural values on consumer visits using normalized variables

**Variable**	**Model (1)**	**Model (2)**	**Model (3)**
Time-related variables			
WHOIMMEDIATE	.86**	—	.93**
	(.000)		(.000)
WHOEXTEND	−.38**	—	−.47**
	(.000)		(.000)
WHOTREND	−.03**	−.04**	−.02**
	(.000)	(.000)	(.000)
TIME	.01**	.01**	.01**
	(.000)	(.000)	(.000)
Interactions for Hofstede cultural dimensions			
Uncertainty avoidance[1] (times) WHOIMMEDIATE	.29**	.29**	.27**
	(.000)	(.000)	(.000)
Long-term orientation[1] (times) WHOIMMEDIATE	−.21**	−.20**	−.24**
	(.006)	(.006)	(.001)
Indulgence[1] (times) WHOIMMEDIATE	−.17*	−.17*	−.18*
	(.030)	(.025)	(.024)
Individualism[1] (times) WHOIMMEDIATE	.28**	.29**	.25**
	(.001)	(.000)	(.002)
Power distance[1] (times) WHOIMMEDIATE	.11	.11	.13
	(.182)	(.172)	(.121)
Masculinity[1] (times) WHOIMMEDIATE	−.06	−.06	−.06
	(.342)	(.346)	(.346)
Uncertainty avoidance[1] (times) WHOEXTEND	−.15**	−.14**	−.13**
	(.000)	(.000)	(.000)
Long-term orientation[1] (times) WHOEXTEND	.25**	.24**	.29**
	(.000)	(.000)	(.000)
Indulgence[1] (times) WHOEXTEND	.20**	.20**	.22**
	(.000)	(.000)	(.000)
Individualism[1] (times) WHOEXTEND	−.02	−.01	−.02
	(.434)	(.763)	(.429)
Power distance[1] (times) WHOEXTEND	−.11**	−.10**	−.15**
	(.000)	(.001)	(.000)
Masculinity[1] (times) WHOEXTEND	.02	.01	.02
	(.519)	(.690)	(.494)
Government closure policies (base: None)			
Limited lockdown policy	−.42**	−.52**	−.42**
	(.000)	(.000)	(.000)
Moderate lockdown policy	−.82**	−.89**	−.85**
	(.000)	(.000)	(.000)
Strict lockdown policy	−1.65**	−1.73**	−1.67**
	(.000)	(.000)	(.000)
Contact tracing (base: None)			
Limited contact tracing	.08**	.07	.06
	(.008)	(.148)	(.078)
Comprehensive contact tracing	.02	.06	.04
	(.465)	(.249)	(.189)
Controls (other)			
CDGR of cases over one week[1]	−.02	−.04**	—
	(.115)	(.005)	
CDGR of deaths over one week[1]	−.09**	−.10**	—
	(.000)	(.000)	
Number of daily cases per capita[1]	—	—	−.13**
			(.000)
Number of daily deaths per capita[1]	—	—	.03
			(.057)
GDP per capita[1]	−.04*	—	.02
	(.045)		(.392)
Press freedom[1]	−.00	—	.00
	(.840)		(.922)
Controls for (six) Hofstede cultural dimensions[1]	yes	—	yes
Controls for country fixed effects	no	yes	no
Constant	.37**	.58**	.41**
	(.000)	(.000)	(.000)
**N**	3,078	3,078	3,078
**R^2**	.65	.66	.65
**Adj. R^2**	.64	.66	.64

Note: *p*−values in parentheses; * $p < .05$, ** $p < .01$; [1] normalized.

Figure W1 – Predicted visits to shopping destinations before and after WHO's announcement of the COVID−19 outbreak as a pandemic

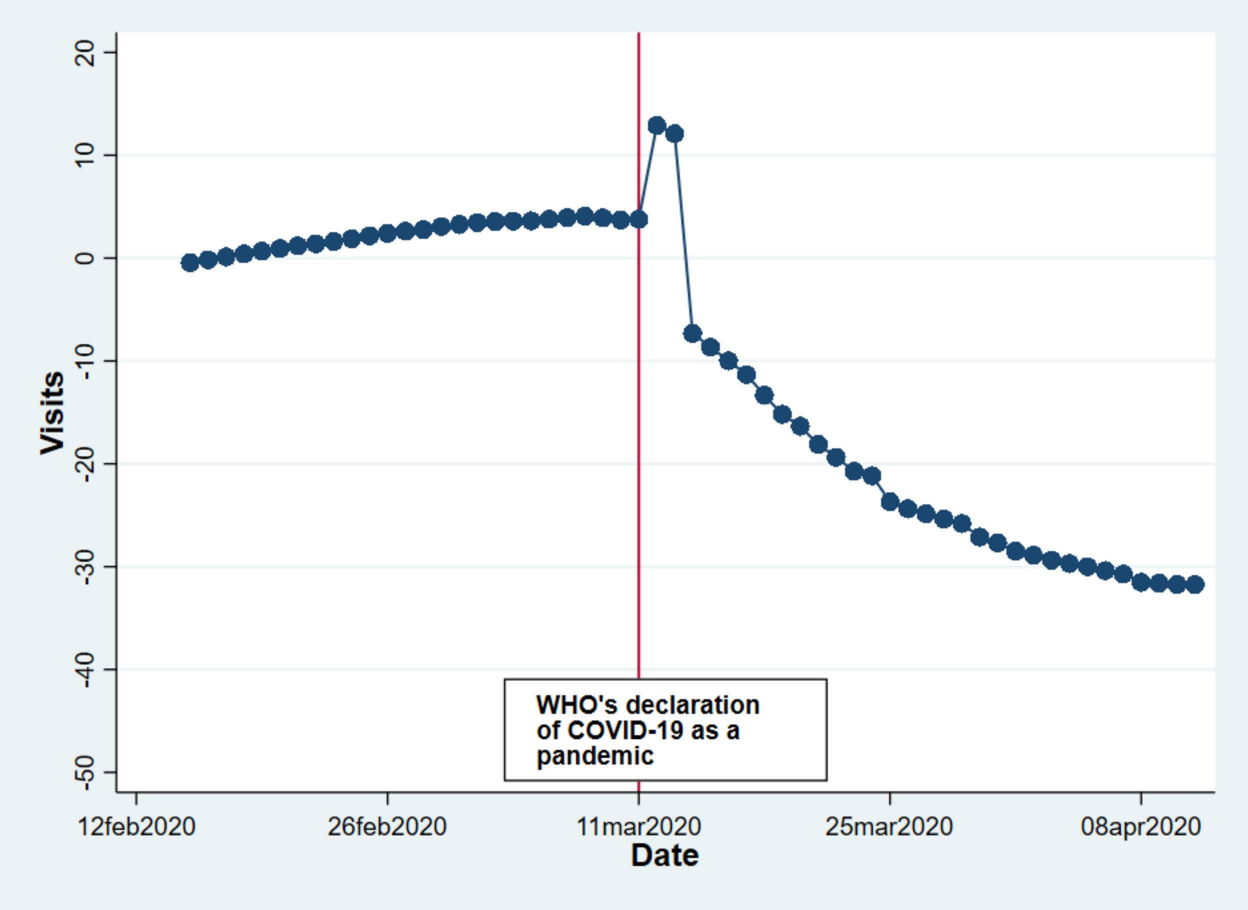

Note: The figure illustrates average predicted visits across 54 countries and is based on the results from Model (1) of Table 1 in the manuscript; the vertical (red) line corresponds to the date that the WHO announced the COVID−19 outbreak as a pandemic.

Figure W2 – Comparison of immediate effects of cultural values on consumer stockpiling due to WHO's announcement of COVID−19 outbreak as a pandemic

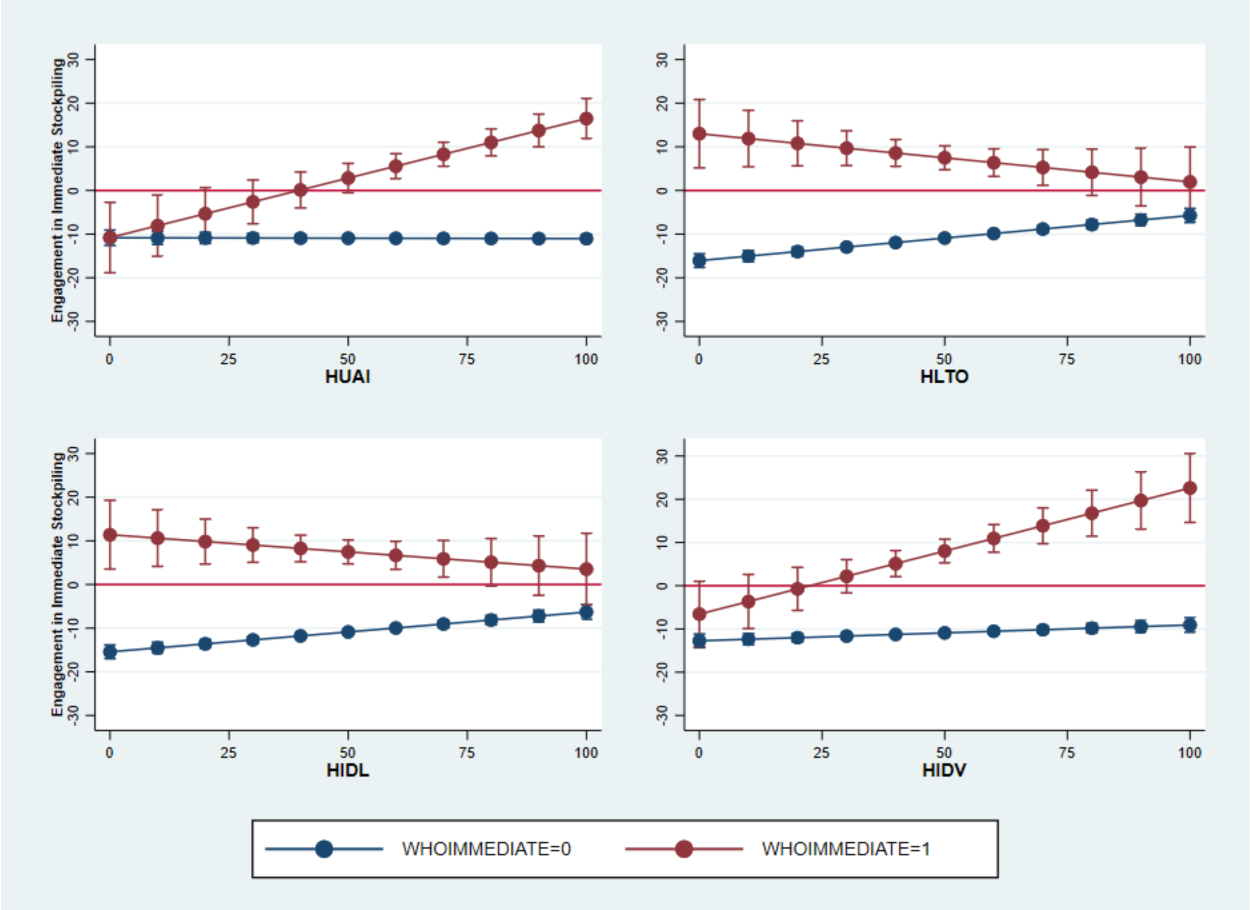

Note: Vertical lines represent 95% confidence interval; predicted values are estimated while setting all values at their means; across all countries, in the two days following the WHO's announcement, consumers engaged in stockpiling, and that this phenomenon was particularly pronounced in countries of high uncertainty avoidance, low long-term orientation, low indulgence, and high individuality.
